# First stem cell transplantation to regenerate human lung

**DOI:** 10.1007/s13238-017-0498-z

**Published:** 2018-01-04

**Authors:** Si Wang, Jun Wu, Guang-Hui Liu

**Affiliations:** 1 National Laboratory of Biomacromolecules, CAS Center for Excellence in Biomacromolecules, Institute of Biophysics, Chinese Academy of Sciences, Beijing, 100101 China; 2 University of Chinese Academy of Sciences, Beijing, 100049 China; 3 National Clinical Research Center for Geriatric Disorders, Xuanwu Hospital of Capital Medical University, Beijing, 100053 China; 4 Department of Molecular Biology, University of Texas Southwestern Medical Center, Dallas, TX 75390 USA; 5 Hamon Center for Regenerative Science and Medicine, University of Texas Southwestern Medical Center, Dallas, TX 75390 USA

Lung-related diseases are the third-leading cause of human death throughout the world. Lethal lung diseases such as chronic obstructive pulmonary disease (COPD), pulmonary fibrosis and bronchiectasis are characterized by irreversible, progressive damage of the lung tissue. The ability to regenerate human lung tissue, if successful, would constitute a breakthrough in modern medicine. Despite years of research, however, currently there is no effective strategy to regenerate lost bronchioles or alveoli in humans. Stem cell-based regenerative medicine holds great potential for combating tissue damage in lung diseases and other disorders by providing unlimited materials for transplant (Liu et al., 2012; Liu et al., 2014; Wu and Izpisua Belmonte, 2016; Shi et al., 2017; Yang et al., 2017). Induced pluripotent stem cells (iPSCs) could be the source of cells for autologous transplantation. iPSC has been successfully differentiated to alveolar and airway lineage (Huang et al., 2014; McCauley et al., 2017). It remains unknown however, whether these iPSC-derivatives can form lung tissue *in vivo*, and/or functionally contribute to gas-blood air exchange. In addition, undifferentiated iPSCs in the differentiation culture generate safety concerns. On the other hand, somatic stem cells or progenitors—if can be identified, isolated and expanded—could offer a better and safer choice for regenerative medicine.

Different types of lung stem/progenitor cells have been identified and extensively studied in rodents (Hogan et al., 2014; Vaughan et al., 2015; Zuo et al., 2015). To date, however, human lung stem/progenitor cells remain elusive (Kajstura et al., 2011). Given the vast differences between human and mouse in the development, tissue architecture (e.g., lobulation and branching pattern) and cell composition of the respiratory system, existing information in the rodents may not be directly translated into humans. Therefore, additional efforts are needed to identify and characterize human lung stem/progenitor cells.

In this issue of *Protein & Cell*, Ma et al. identified a previously unknown population of adult human lung stem cells, which were successfully used for generating functional human lung air exchanging units following their transplantation to a mouse model and also in a pilot human clinical trial (Ma et al. 2018).

The putative adult human lung stem cells discovered by Ma et al. are located to the bottom of rugae in 2–4 order airway and could be distinguished from airway basal cells (BCs) by SOX9 expression. SOX9 is a known marker for embryonic lung progenitor cells and also stem/progenitor cells of other tissues such as liver and gut. Interestingly, in gut the SOX9+ progenitors reside in the crypt, an invaginated structure similar to the airway rugae. From a trace amount of bronchoscopic brush-off lung tissues, the authors isolated SOX9+ BCs and expanded them *in vitro* for a long time. Clonally derived SOX9+ BCs were carefully characterized and then transplanted into injured mouse lung. Following transplantation, SOX9+ BCs could give rise to both human bronchiolar and alveolar epithelium *in vivo*, demonstrating their generative potential. It is worth noting that SOX9+ BCs could be easily isolated from both normal and diseased patients by routine, bleeding-free bronchoscopic brushing with a high success rate. Also the culture method the authors developed to expand SOX9+ BCs *in vitro* is pivotal for producing sufficient cells for transplantation. The culture method for adult the putative human lung stem cells developed in this study has certain advantage over the bronchiolar/alveolar organoid culture method reported previously (Nichane et al., 2017), in that it enables the production of a large number of relatively pure population of cells from as few as one single cell, which can maintain their developmental potential even in a feeder-free culture condition.

By transplanting the *in vitro* expanded cells into immune-deficient mice, Ma et al. generated a chimeric lung with both human and mouse tissues. The regenerated human alveoli established intact vasculature system and were functional. However, human type II alveoli cells were not generated, which is likely due to the limited potency of the human cells and/or species difference. More importantly, in this study, for the first time the authors explored the clinical feasibility of autologous SOX9+ BC transplantation to treat two patients with bronchiectasis. The results showed recovery of patients’ pulmonary function and improvement of bronchi dilation on CT over time. Although it is a very small sample size, the results of this pilot trial are consistent with their observation in the mouse model, and pave the road for a large-scale controlled clinical study in the future. Interestingly, the authors also showed that the anti-lung fibrosis drug Pirfenidone could boost stem cell transplantation efficiency in a TGFβ-dependent manner, suggesting the possible combination of traditional chemical pills and stem cell transplantation for combating lung diseases.

Although Ma et al. established an applicable system for human lung regeneration, there are still several issues remain unsolved—for instance, whether the stem cell transplantation therapy works well in treating other lung-related diseases, such as emphysema or idiopathic pulmonary fibrosis, and the long term effect of stem cell transplantation remains unexplored.

In summary, the identification and culture of human lung stem/progenitor cells reported in this study hold great potential for developing regenerative medicine therapy for treating lung diseases. Considering chronic lung diseases are life threatening with only palliative treatments available, the significance of lung stem/progenitor cell-based transplantation would be tremendous, which provides exciting translational opportunities ahead.

## References

[CR1] Hogan BL, Barkauskas CE, Chapman HA, Epstein JA, Jain R, Hsia CC, Niklason L, Calle E, Le A, Randell SH (2014). Repair and regeneration of the respiratory system: complexity, plasticity, and mechanisms of lung stem cell function. Cell Stem Cell.

[CR2] Huang SX, Islam MN, O’Neill J, Hu Z, Yang YG, Chen YW, Mumau M, Green MD, Vunjak-Novakovic G, Bhattacharya J (2014). Efficient generation of lung and airway epithelial cells from human pluripotent stem cells. Nat Biotechnol.

[CR3] Kajstura J, Rota M, Hall SR, Hosoda T, D’Amario D, Sanada F, Zheng H, Ogorek B, Rondon-Clavo C, Ferreira-Martins J (2011). Evidence for human lung stem cells. N Engl J Med.

[CR4] Liu GH, Qu J, Suzuki K, Nivet E, Li M, Montserrat N, Yi F, Xu X, Ruiz S, Zhang W (2012). Progressive degeneration of human neural stem cells caused by pathogenic LRRK2. Nature.

[CR5] Liu GH, Suzuki K, Li M, Qu J, Montserrat N, Tarantino C, Gu Y, Yi F, Xu X, Zhang W (2014). Modelling Fanconi anemia pathogenesis and therapeutics using integration-free patient-derived iPSCs. Nat Commun.

[CR100] Ma Q, Ma Y, Dai X, Ren T, Fu Y, Liu W, Han Y, Wu Y, Cheng Y, Zhang T et al (2018) Regeneration of functional alveoli by adult human SOX9+ airway basal cell transplantation. Protein Cell. 10.1007/s13238-018-0506-y 10.1007/s13238-018-0506-y PMC5829276 29344809

[CR6] McCauley KB, Hawkins F, Serra M, Thomas DC, Jacob A, Kotton DN (2017). efficient derivation of functional human airway epithelium from pluripotent stem cells via temporal regulation of Wnt signaling. Cell Stem Cell.

[CR7] Nichane M, Javed A, Sivakamasundari V, Ganesan M, Ang LT, Kraus P, Lufkin T, Loh KM, Lim B (2017). Isolation and 3D expansion of multipotent Sox9(+) mouse lung progenitors. Nat Methods.

[CR8] Shi Y, Inoue H, Wu JC, Yamanaka S (2017). Induced pluripotent stem cell technology: a decade of progress. Nat Rev Drug Discov.

[CR9] Vaughan AE, Brumwell AN, Xi Y, Gotts JE, Brownfield DG, Treutlein B, Tan K, Tan V, Liu FC, Looney MR (2015). Lineage-negative progenitors mobilize to regenerate lung epithelium after major injury. Nature.

[CR10] Wu J, Izpisua Belmonte JC (2016). Stem Cells: A Renaissance in Human Biology Research. Cell.

[CR11] Yang J, Li J, Suzuki K, Liu X, Wu J, Zhang W, Ren R, Chan P, Izpisua Belmonte JC, Qu J (2017). Genetic enhancement in cultured human adult stem cells conferred by a single nucleotide recoding. Cell Res.

[CR12] Zuo W, Zhang T, Wu DZ, Guan SP, Liew AA, Yamamoto Y, Wang X, Lim SJ, Vincent M, Lessard M (2015). p63(+)Krt5(+) distal airway stem cells are essential for lung regeneration. Nature.

